# Combined light- and heat-induced shape memory behavior of anthracene-based epoxy elastomers

**DOI:** 10.1038/s41598-020-77246-0

**Published:** 2020-11-19

**Authors:** Yuzhan Li, Monojoy Goswami, Yuehong Zhang, Tuan Liu, Jinwen Zhang, Michael R. Kessler, Liwei Wang, Orlando Rios

**Affiliations:** 1 Energy and Transportation Science Division, Oak Ridge National Laboratory, Oak Ridge, TN 37831 USA; 2 Chemical Sciences Division, Oak Ridge National Laboratory, Oak Ridge, TN 37831 USA; 3 College of Bioresources Chemical and Materials Engineering, Shaanxi University of Science and Technology, Xi’an, 710021 China; 4 School of Mechanical and Materials Engineering, Washington State University, Pullman, WA 99164 USA; 5 Department of Mechanical Engineering, North Dakota State University, Fargo, ND 58108 USA; 6 Department of Materials Science and Engineering, The University of Tennessee, Knoxville, TN 37996 USA

**Keywords:** Polymers, Coarse-grained models

## Abstract

The development of multi-stimuli-responsive shape memory polymers has received increasing attention because of its scientific and technological significance. In this work, epoxy elastomers with reversible crosslinks are synthesized by polymerizing an anthracene-functionalized epoxy monomer, a diepoxy comonomer, and a dicarboxylic acid curing agent. The synthesized elastomers exhibit active responses to both light and heat enabled by the incorporated anthracene groups. When exposed to 365 nm UV light, additional crosslinking points are created by the photo-induced dimerization of pendant anthracene groups. The formation of the crosslinking points increases modulus and glass transition temperature of the elastomers, allowing for the fixation of a temporary shape at room temperature. The temporary shape remains stable until an external heat stimulus is applied to trigger the scission of the dimerized anthracene, which reduces the modulus and glass transition temperature and allows the elastomers to recover their original shapes. The effects of external stimuli on the thermal and dynamic mechanical properties of the elastomers are investigated experimentally and are correlated with molecular dynamics simulations that reveal the changes of structure and dynamics of the anthracene molecules and flexible chains.

## Introduction

Shape memory polymers (SMPs) are smart materials that can recover shapes from a deformed state to the original state when triggered by external stimuli^1,2^, such as heat^3,4^, light^5,6^, pH^7^, moisture^8,9^, electric fields^10,11^, or magnetic fields^12,13^. SMPs usually possess physically or chemically crosslinked networks, which determine the original shape of the material, and switching segments, which are crucial for shape fixation and shape recovery because of their reversible phase transitions^14^. In the shape programming process, the switching segments are brought into a softened state so that the material can be deformed to adopt a temporary shape. Subsequent solidification of the switching segments results in fixation of the temporary shape. In the shape recovery process, the switching segments are softened for the polymer network to gain entropy and return to the original shape.

Compared to heat-induced shape memory behavior, light-controlled shape fixation or recovery show some advantages, such as remote and spatial control, where the shape changes can be activated from a long distance and on a specific location of the material^15^. These unique features make light-controlled SMPs excellent candidates for applications where temperature change is not desirable, such as biomedical applications. Several light-controlled SMPs have been synthesized and reported. The light-induced shape memory behavior of these materials is mostly based on the photo-thermal effect of incorporated fillers. Light-sensitive molecules or particles, such as azobenzene^16,17^, gold nanoparticles^18,19^, and carbon nanotubes^20,21^ are either chemically incorporated into the polymer structures or physically dispersed within the polymer matrix. They strongly absorb light and convert it to heat, raising the temperature of the material to activate the switching segments. In this approach, heat remains the ultimate stimulus to enable shape fixation and recovery^22^. Another mechanism of light-controlled shape memory effect is based on photochemical reactions, such as reversible photo-crosslinking^23–25^. Anthracene molecules exhibit a reversible dimerization reaction enabled by a [4 + 4] cycloaddition when exposed to UV light with wavelengths higher than 300 nm and a scission of the crosslinked dimers when exposed to UV light with wavelengths lower than 300 nm or temperature above 120 °C^26^. Based on this mechanism, anthracene has been incorporated into a variety of polymers as pendant groups to realize light-controlled shape memory behavior, including poly(l-lactide) (PLA)^27^, poly(ethylene glycol) (PEG)^28^, poly(d,l-lactide) (PDLLA)^29^, poly(tetramethylene oxide) glycol (PTMEG)^30^. These anthracene-functionalized polymers showed effective shape fixation and recovery when irradiated by 365 nm and 254 nm UV light, respectively, which was ascribed to the photodimerization and scission of the pendant anthracene groups. The reversible anthracene dimerization has also been used as a strategy to tune the mechanical properties of thermoplastic fibers that are produced using a high-throughput melt blowing process^31^. However, the incorporation of anthracene into epoxy networks for the design of shape memory epoxy resins remains largely unexplored. Radl et al. synthesized epoxy prepolymers bearing anthracene groups. When exposed to UV light, the prepolymers formed a network structure because of the light-induced dimerization of the anthracene groups. Heating to temperatures up to 130 °C led to thermally induced cleavage of the photodimers^32^. This reversible covalent network formation was utilized for the development of mendable epoxy materials. Similarly, Hughes et al. reported the synthesis of anthracene-based diamine crosslinkers, which were used to react with different epoxy monomers for the development of light-healable crosslinked epoxy polymers^33^. However, in these previously reported systems, the reversible anthracene dimerization was mainly used as a strategy to realize self-healing behavior.

In this work, we report the development of anthracene-based epoxy elastomers with reversible crosslinks and show a combined light- and heat-induced shape memory behavior enabled by the covalently incorporated anthracene groups. An anthracene-functionalized epoxy monomer, 9-anthracenemethoxyl glycidyl ether (AN), was synthesized and cured with 1,4-butanediol diglycidyl ether (BDE) and sebacic acid (SA) to form soft, amorphous epoxy elastomers with pendant anthracene groups. The concentration of anthracene molecules within the system was controlled by varying the molar ratio of AN, BDE, and SA. The influence of the incorporated anthracene groups on the thermomechanical and shape memory properties of the resulting networks are investigated using both thermal analysis and molecular dynamics simulations.

## Results and discussion

### Design and synthesis of epoxy elastomers

**Figure 1:**
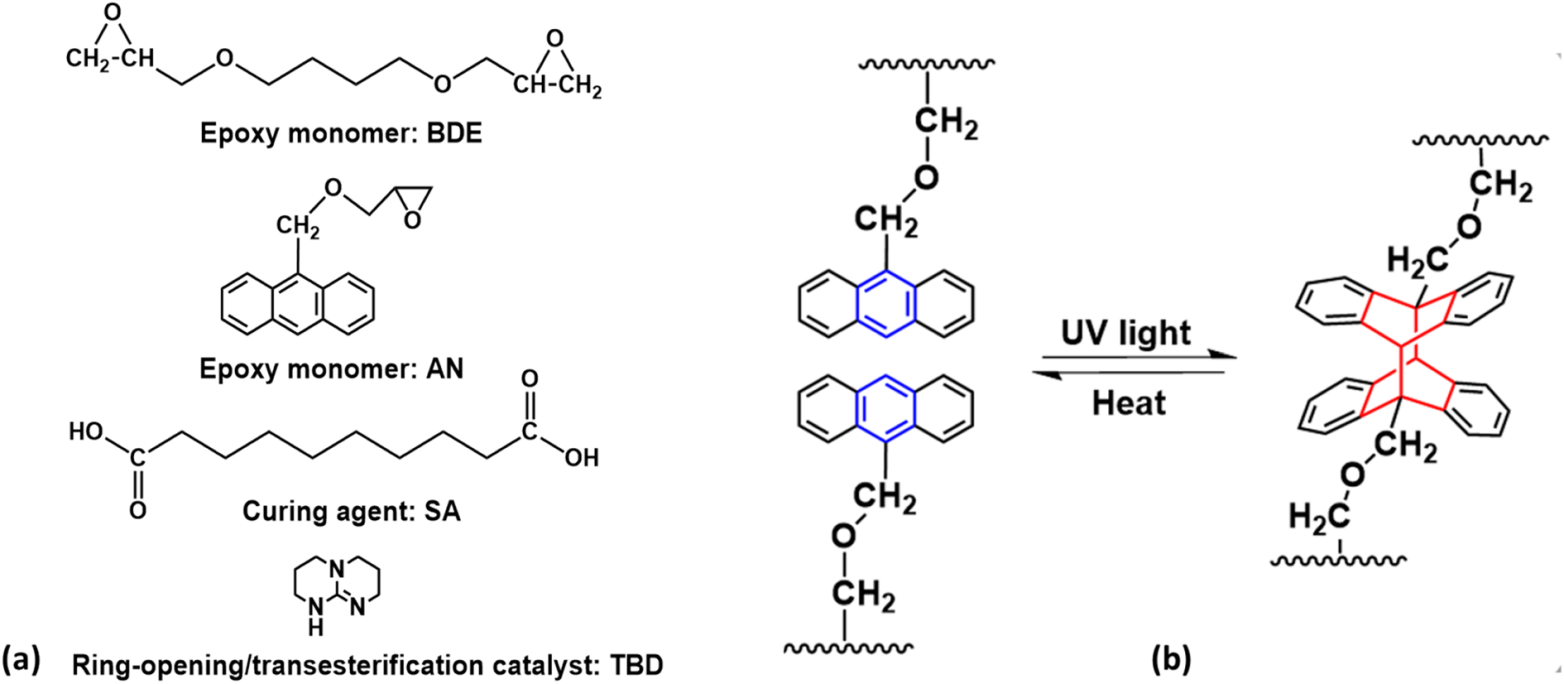
Design and synthesis of anthracene-based epoxy elastomers. (**a**) Chemical structure of epoxy monomers, dicarboxylic acid curing agent, and catalyst. (**b**) Schematic illustration of reversible crosslinking reaction of anthracene molecules.

**Figure 2:**
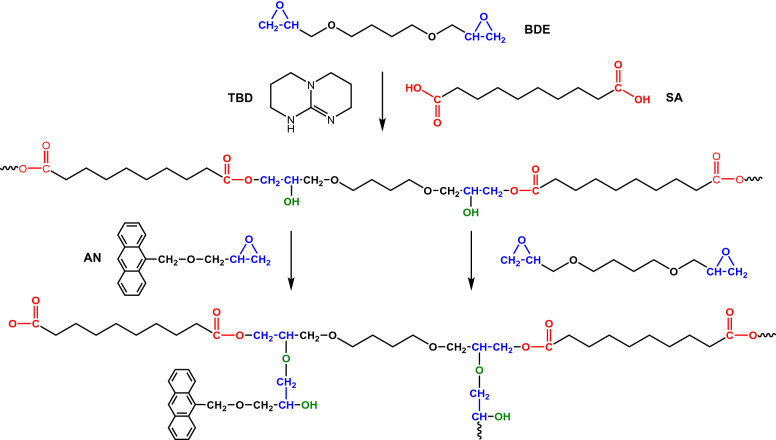
Curing reaction of the epoxy elastomers showing the incorporation of anthracene molecules as pedant groups in the crosslinking networks.

The chemical structure of the components for the synthesis of anthracene-based epoxy elastomers is shown in Fig. 1a. BDE and SA were selected because of their difunctionality and flexible chain structures, which facilitates the formation of an amorphous elastomeric network with low T_g_. A monofunctional AN was synthesized using a simple reaction between 9-anthracenemethanol and epichlorohydrin (Figure S1 in SI). By using the monofuntional epoxy monomer, anthracene molecules were incorporated into the network as pendant groups, which is critical to enabling the reversible photo-crosslinking reaction, as shown in Fig. 1b. In addition, the monofunctionality allowed for the preparation of elastomers with high concentration of anthracene molecules without significantly increasing T_g_ of the material. The soft, amorphous elastomer system also allowed us to investigate the intrinsic influence of anthracene dimerization and scission on the properties of the materials. Epoxy elastomers with different concentration of anthracene groups were prepared by varying the molar ratio between AN and BDE while maintaining an epoxy/acid molar ratio of 2:1. Detailed formulation of the epoxy elastomers is summarized in Table 1. The reactions involved in the curing process are shown in Fig. 2. During the curing process, hydroxyl groups were generated from the reaction between epoxy and carboxylic acid and acted as crosslinking points in the elastomeric network. The epoxy/acid ratio was kept at 2:1 to ensure a full consumption of carboxylic acid groups by the epoxy monomer as the remaining acid groups can react with hydroxyl groups to form water, resulting in trapped air bubbles in the elastomers.

**Table 1:** Formulation of epoxy elastomers with different anthracene concentration.

	BDE (mol%)	AN (mol%)	SA (mol%)
EE-AN-0	10	0	5
EE-AN-20	10	4	6
EE-AN-40	10	12	8

### Thermal and dynamic mechanical properties

**Figure 3:**
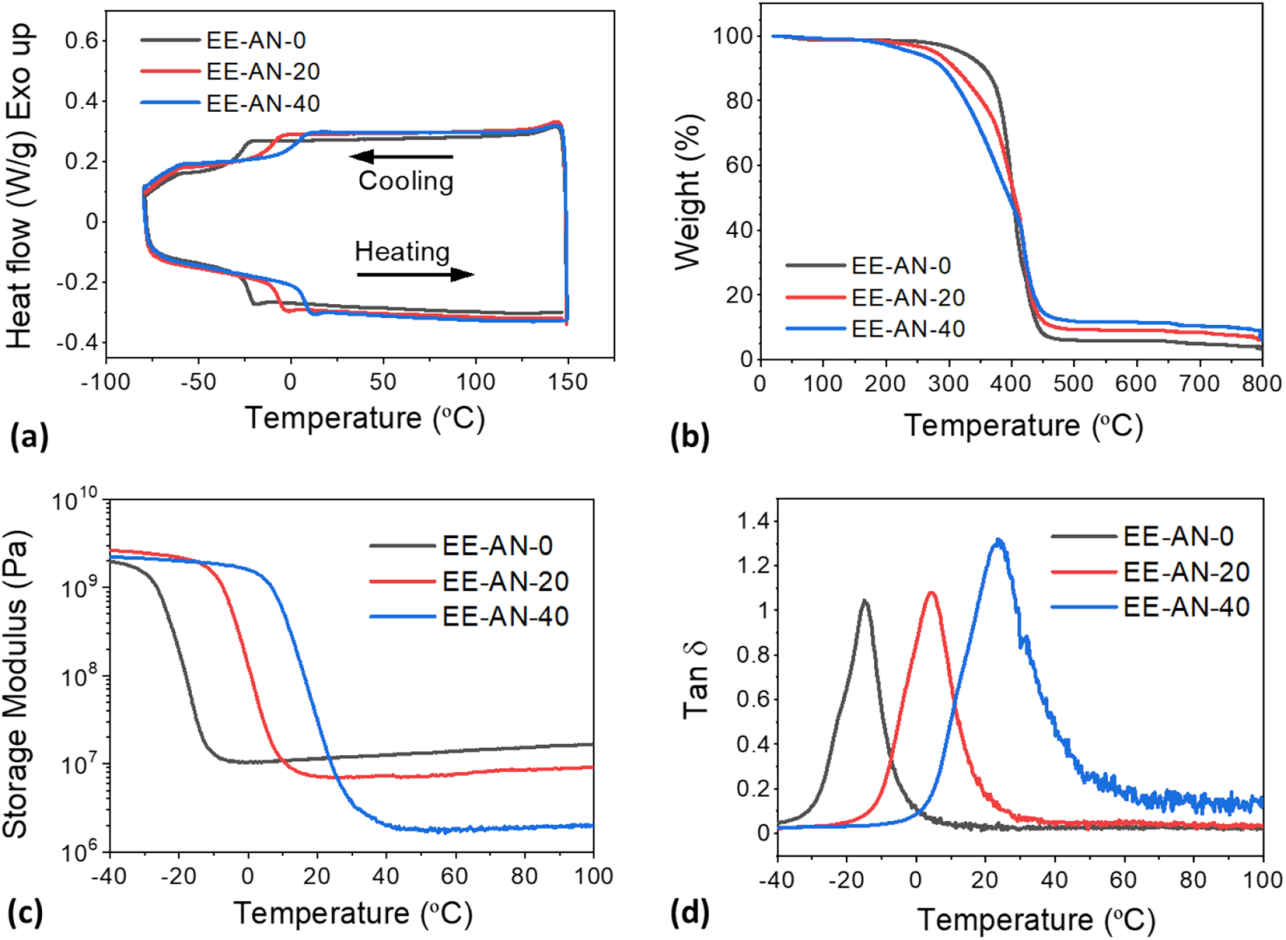
Effect of anthracene concentration on thermal and dynamic mechanical properties of as-prepared epoxy elastomers. (**a**) First cooling and second heating DSC scans. (**b**) Change of weight percentage as a function of temperature. (**c**) Storage moduli as a function of temperature. (**d**) Dissipation factor as a function of temperature.

Anthracene is a polycyclic aromatic molecule with three fused benzene rings. Because of its rigid structure, the incorporation of anthracene significantly influenced properties of the epoxy elastomers. Figure 3a shows first cooling and second heating DSC scans of the epoxy elastomers with different anthracene concentration. The elastomer without anthracene groups (EE-AN-0) showed a T_g_ of − 23.9 °C because of the lightly crosslinked structure and the flexible chains of BDE and SA molecules. After the incorporation of AN, the T_g_ of the material increased to − 7.6 °C for EE-AN-20 and 6.6 °C for EE-AN-40, respectively. It is noteworthy that the incorporation of AN decreased the crosslink density of the elastomers due to its monofunctional nature, which was expected to reduce T_g_ of the materials. However, the rigid molecular structure of AN predominantly influenced mobility of the polymer chains and resulted in an increase of T_g_. The incorporation of AN also showed influences on thermal stability of the elastomers, as indicated by the TGA curves in Fig. 3b. The degradation temperature (T_d_) of the elastomers was determined using the temperature at which the material showed 5% weight loss. Although anthracene is characterized by a rigid aromatic structure, the introduction of anthracene resulted in a decrease in T_d_ of the elastomers, indicating that crosslinking density predominantly influenced the thermal stability of the epoxy elastomers. As the anthracene concentration increased from 0 to 40 mol%, T_d_ of the elastomer decreased from 318 to 240 °C, attributed to the highly branched structure due to the incorporation of the monofunctional anthracene monomer. When comparing the residual weight percent of the epoxy elastomers, EE-AN-40 exhibited the highest values, indicating a high concentration of anthracene molecules, as char formation is promoted by aromatic structures. The structure–property relationships of the epoxy elastomers were further investigated using DMA. The storage modulus and dissipation factor of the elastomers are shown in Fig. 3c,d. The T_g_ of the elastomers increased with increasing AN concentration as indicated by the shifted tan δ peak, similar to that was observed in the DSC experiment. As mentioned previously, the incorporation of AN reduced crosslinking density of the materials because of the monofunctional AN monomer. This was confirmed by the lower rubbery modulus of the elastomers with higher AN concentration. The crosslinking density was calculated based on the rubber elasticity theory, in which the rubbery plateau modulus is inversely proportional to the molecular weight between two successive entanglements. The detailed thermal and dynamic mechanical properties of the epoxy elastomers are summarized in Table 2.

**Table 2:** Thermal and dynamic mechanical properties of as-prepared epoxy elastomers with different concentrations of anthracene.

Sample	T_g_ (°C)^a^	T_g_ (°C)^b^	T_d_ (°C)	Rubbery modulus (MPa)^c^	Crosslinking density (mol/m^3^)
EE-AN-0	− 23.9	− 14.9	318	12.9	2007
EE-AN-20	− 7.6	4.1	277	7.9	1142
EE-AN-40	6.6	23.6	240	1.9	264

^a^Measured from the midpoint of the step change in the second heating DSC scan.

^b^Measured from the peak of the tan δ curve.

^c^Measured from storage modulus at T_g_ + 60 °C.

### Photo-responsive properties

**Figure 4:**
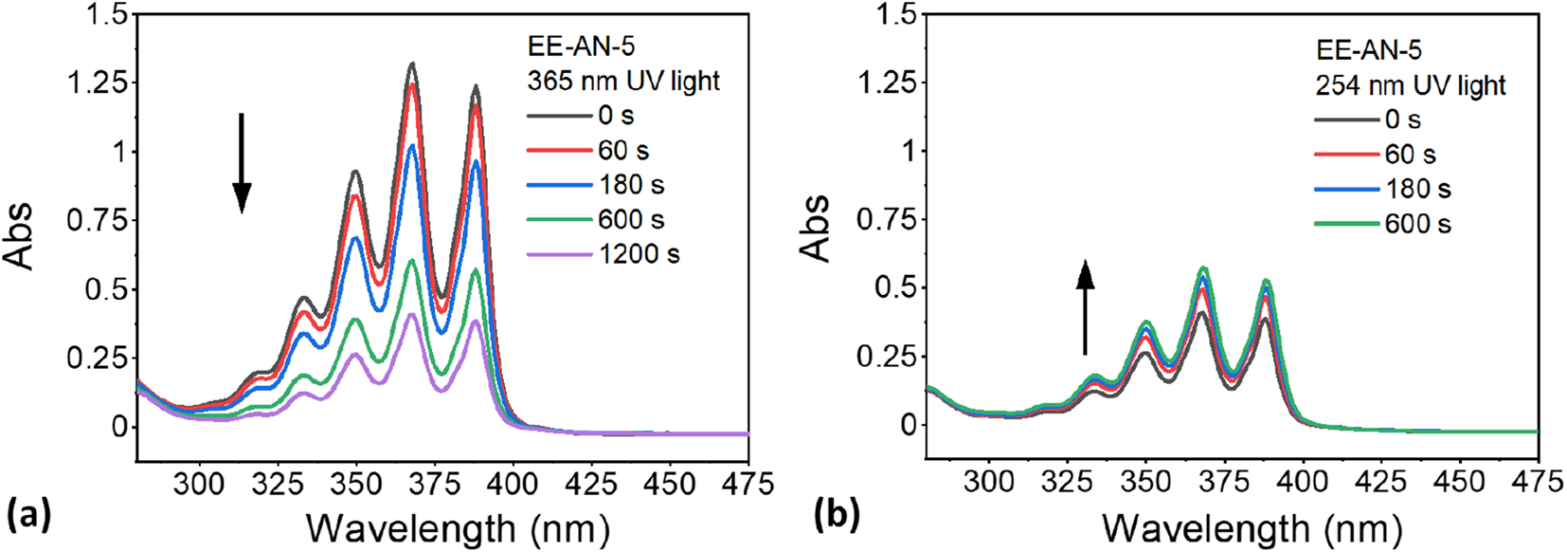
Photo-responsive behavior of the synthesized epoxy elastomer enabled by the reversible anthracene photodimerization. (**a**) UV–vis absorption spectra of an as-prepared elastomer after the exposure of 365 nm UV light for different time durations. (**b**) After the UV exposure at 365 nm, UV–vis absorption spectra of the elastomer after the exposure of 254 nm UV light for different time durations.

**Figure 5:**
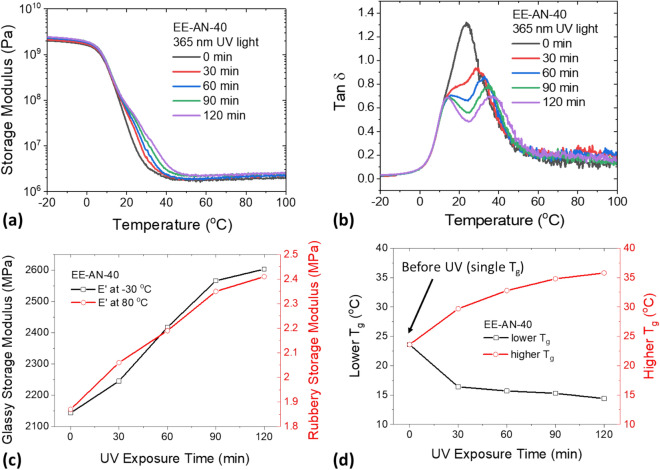
Effect of photodimerization of anthracene on the dynamic mechanical properties of epoxy elastomer (EE-AN-40). (**a**) Storage modulus as a function of temperature. (**b**) Dissipation factor as a function of temperature. (**c**) Glassy and rubbery moduli as a function of UV exposure time. (**d**) Glass transition temperatures as a function of UV exposure time.

As mentioned in previous sections, anthracene molecules were incorporated into the soft epoxy network as pendent groups to improve their mobility and facilitate their reversible dimerization. To investigate the reversible photo-crosslinking reaction, a thin film sample was prepared between two quartz plates. This sandwiched setup avoided excessive exposure of the epoxy film to oxygen, which is known to influence reversibility of the anthracene dimerization process^34^. UV–Vis spectra of the material after UV exposure at different wavelengths and time durations were collected and compared. Our initial attempt on using a thin film sample of EE-AN-40 for the UV–Vis spectroscopy measurement was not successful due to the strong UV absorption coefficient of anthracene and the high concentration of anthracene in the EE-AN-40 formulation. Therefore, the anthracene concentration was reduced to 5 mol% and the thin film elastomer was labeled as EE-AN-5. Figure 4a shows the UV–Vis absorption spectra of the as-prepared EE-AN-5 film under the exposure of 365 nm for different time durations. Before UV exposure, the material showed four strong absorption peaks at 333, 350, 368, and 388 nm, which were attributed to the conjugated bonds of anthracene. The intensity of these peaks gradually decreased with the increasing exposure time of the 365 nm UV light, indicating the photodimerization of the pendant anthracene groups, accompanied by the disappearance of the conjugated bonds through the formation of an eight-membered ring by a [4 + 4] cycloaddition reaction, as schematically illustrated in Fig. 1b. By calculating the decreased absorption intensity at 368 nm, the photodimerization conversion rate of anthracene groups reached to about 70% after irradiation for 1200 s. After the exposure of 365 nm UV light, the epoxy film was exposed to 254 nm UV light for different time durations. UV–Vis spectra of the film were collected and are shown in Fig. 4b. The absorption peaks at 333, 350, 368, and 388 nm increased slightly with the increasing exposure time of 254 nm UV light, indicating scission of the previously dimerized anthracene groups and reformation of the conjugated bonds of anthracene. However, compared to the high photodimerization conversion rate, the scission rate calculated from the increased absorption intensity at 368 nm was relatively low, which was caused by the high stability of the [4 + 4] cycloadduct in solid state and low penetration depth of the 254 nm UV light. The low scission rate was also caused by the change of thermal and mechanical properties of the material after the anthracene dimerization, which is shown in Fig. 5 and is discussed in the following paragraph.

The effect of photodimerization of anthracene on the thermal and mechanical properties of the epoxy elastomers was further investigated using DMA. Sample films prepared using the EE-AN-40 formulation were exposed to 365 nm UV light for different time durations, and then oscillatory tensile tests were performed. Figure 5a shows the change of storage modulus of the elastomer upon the UV exposure. The storage modulus of the material showed active responses to the UV exposure, as shown in Fig. 5c. The glassy storage modulus (measured at − 30 °C) increased from 2150 to 2600 MPa and the rubbery storage modulus (measured at 80 °C) increased from 1.9 to 2.4 MPa after 120 min of the UV exposure. This was attributed to the UV-induced dimerization of the anthracene groups, which transformed from pendant groups to rigid crosslinks, restricting mobility of the soft elastomeric network. An interesting observation is the change of dissipation factors of the material upon UV exposure, as shown in Fig. 5b. A single tan δ peak split into two peaks, indicating the presence of two T_g_s with one lower than the original T_g_ and the other one higher than the original T_g_ (Fig. 5d). This was caused by the incomplete penetration of the UV light to the elastomer film due to the high UV attenuation coefficient of anthracene. The photodimerization was limited on the surface of the material and created a region with a higher T_g_ because of the rigid cycloadduct crosslinks. The formation of the region with a lower T_g_ might be caused by the anthracene groups that were not able to dimerize, whose non-covalent interactions, such as π-π stacking, were disrupted by the cycloaddition of other anthracene molecules. The changes of thermal and mechanical properties of the epoxy film caused by the anthracene dimerization were also believed to be responsible for the low scission rate observed in Fig. 4b. The increased storage modulus and glass transition temperature significantly reduced mobility of the polymer chains, leading to an incomplete scission of the dimerized anthracene.

### Shape memory behavior

**Figure 6:**
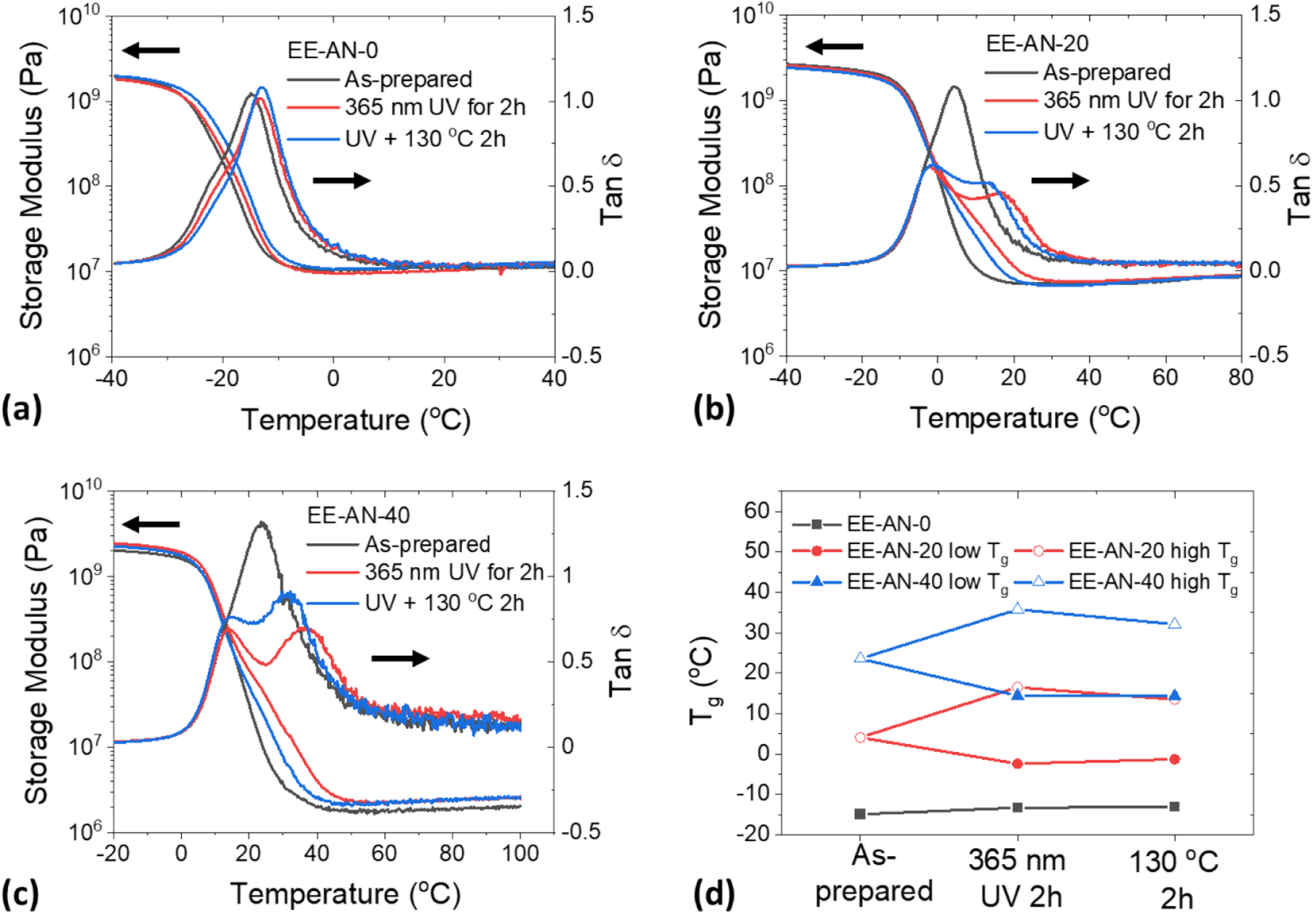
Effect of reversible dimerization of pendant anthracene groups on the properties of epoxy elastomer films. (**a**) EE-AN-0. (**b**) EE-AN-20. (**c**) EE-AN-40. (**d**) Change of glass transition temperatures of epoxy elastomers in response to applied UV light and heat.

In general, a complete shape memory cycle includes a shape fixation process where the polymer chains lose mobility due to the solidification of switching segments and a shape recovery process where the polymer chains re-gain mobility due to the softening of the switching segments. In this study, the UV-induced dimerization of pendant anthracene groups and heat-induced scission of the anthracene dimer were used as the mechanism for shape fixation and recovery, respectively, and were investigated in detail using DMA. Three conditions were compared, including as-prepared films, UV-exposed films, and heated films after the UV exposure. Figure 6 shows the comparison results for all three formulations. The epoxy elastomer without anthracene groups showed almost no change of storage modulus and tan δ curve (Fig. 6a), whereas the elastomers with 20 mol% and 40 mol% anthracene exhibited active responses to UV light and heat. Both samples showed significant changes of storage moduli and tan δ curves after the UV exposure, as shown in Fig. 6b,c (red lines), suggesting the formation of additional crosslinks by the anthracene cycloadduct. However, exposure of the materials to high temperature reserved the process and drove the storage moduli and tan δ curves back to the original ones, which indicates a reduction of crosslinks due to the scission of the dimerized anthracene (blue lines in Fig. 6b,c). Compared to EE-AN-20, the elastomer with a higher anthracene concentration (EE-AN-40) exhibited a more distinct split of T_g_ (Fig. 6d).

**Figure 7:**
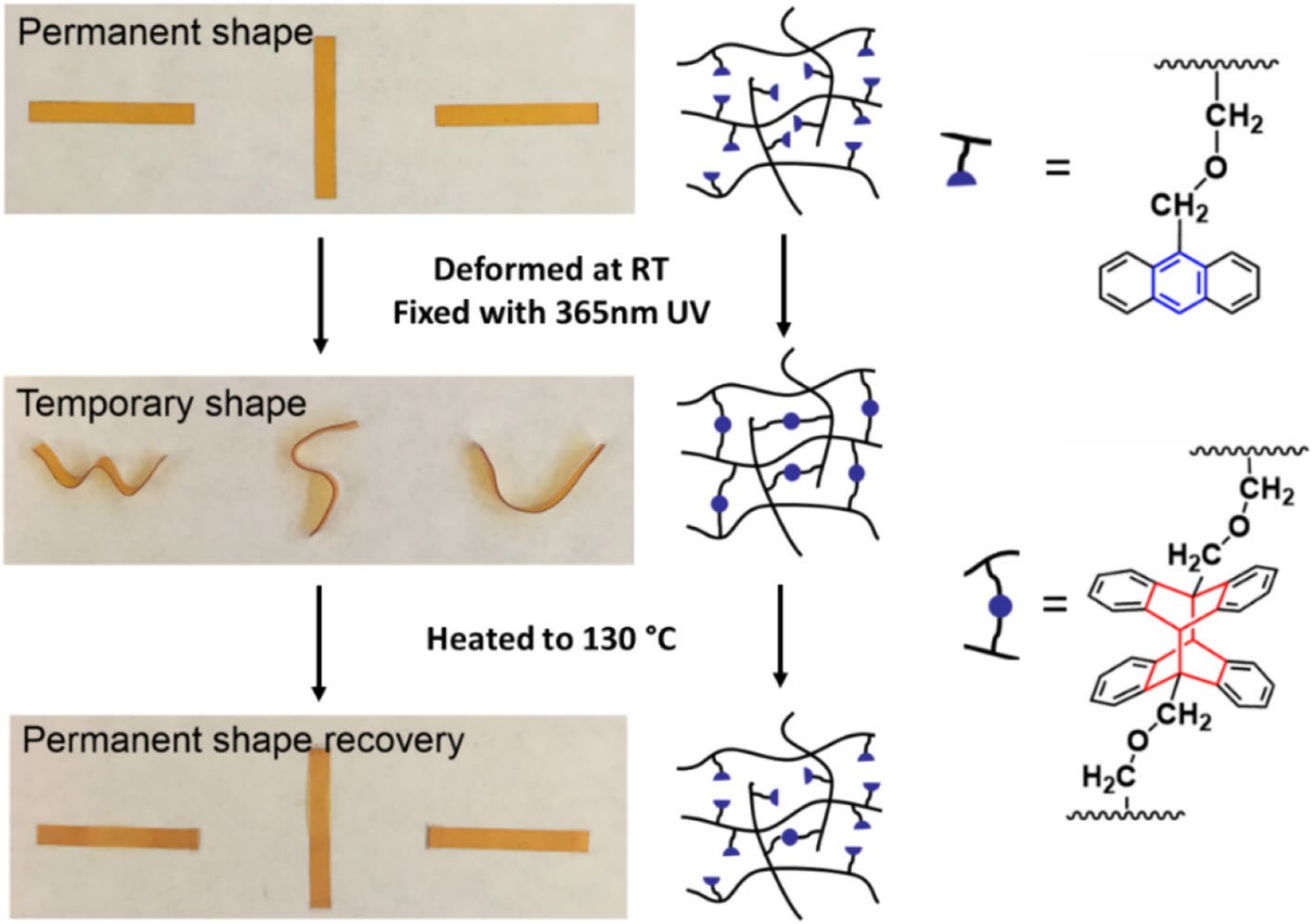
Shape memory behavior of anthracene-based epoxy elastomer enabled by UV-induced shape fixation and heat-induced shape recovery.

Taking advantage of the reversible dimerization of anthracene, shape memory behavior of the epoxy elastomer was realized. Figure 7 illustrates a UV-induced shape fixation process and a heat-induced shape recovery process of the EE-AN-40 epoxy elastomer. EE-AN-40 was chosen because the material is in a rubbery state at room temperature, allowing for convenient shape changing. However, upon UV exposure, the material transformed into a glassy state, allowing for the shape fixation. As shown in Fig. 7, flat strips of EE-AN-40 were deformed into different shapes at room temperature followed by UV exposure at 365 nm for 2 h. After the removal of external force, the strips showed stable temporary shapes. When subjected to heat at 130 °C, the strips rapidly recovered their original flat shapes (Video S1 in SI). It is worth mentioning that while the heat-induced shape recovery of the epoxy film was successfully demonstrated, the high recovery temperature may limit its use in applications where high temperatures are undesirable. However, we anticipate that by slightly heating the 365 nm UV exposed material above its glass transition temperature (around 30 °C, close to body temperature), the scission rate of the dimerized anthracene molecules can be increased, which may enable a light-trigger shape recovery that is suitable for some biomedical applications, such as self-tightening stitches envisioned by Lendlein et al.

### MD simulation

**Figure 8:**
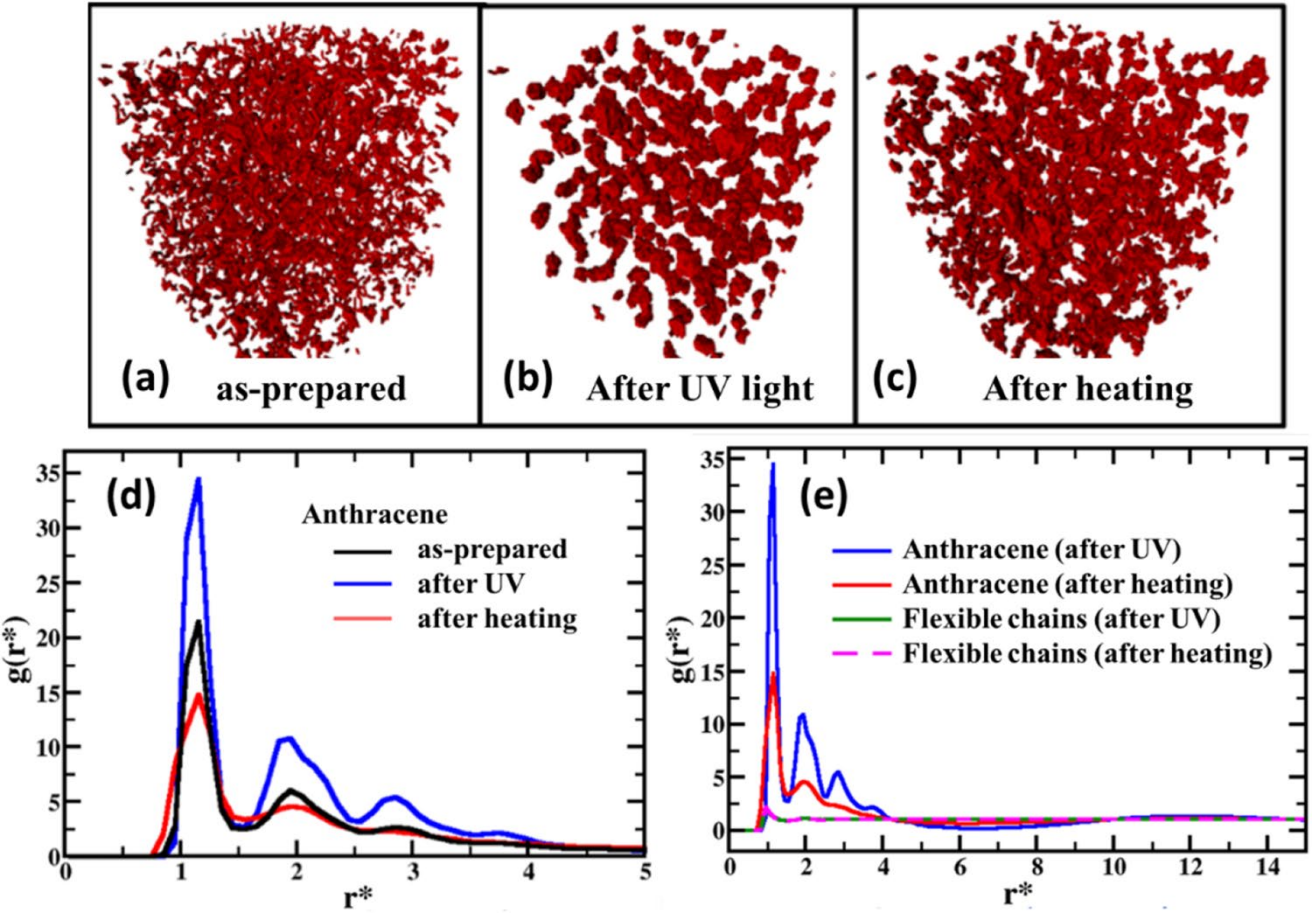
Structure of rigid anthracene molecules and flexible chains of the elastomers from coarse-grained MD simulations. (**a**–**c**) Simulation snapshots of the anthracene molecules under as-prepared, UV-irradiated, and heated conditions. (**d**) Radial distribution function (RDF) of anthracene molecules under different simulating conditions. (**e**) RDF of rigid anthracene molecules and flexible chains before and after heating.

Coarse-grained MD simulations were carried out to understand the effect of external stimuli on the molecular structure and dynamics of the anthracene-based epoxy elastomers. As described in the experimental section, the interaction strength between anthracene molecules was systematically increased to enable bond formation that represents the formation of physical crosslinks, simulating the effect of UV-induced cycloaddition. It must be noted that simulating ‘chemical reactions’ is not possible in coarse-grained MD models, especially the Kremer-Grest bead-spring model. Therefore, physical crosslinking between the pendant side chains of the final polymer structure, shown in Fig. 2 (bottom panel), are considered in these simulations. The reverse cooling process was simulated by increasing the system temperature from T* = 1.0 to T* = 2.0 for first 1 million steps and then reducing the temperature to T* = 1.5 for next 4 million steps. Figure 8a–c shows the simulation snapshots of the anthracene molecules under different simulating conditions. A random spread of anthracene molecules was observed for the as-prepared system (Fig. 8a), which turned into tightly agglomerated smaller domains after UV irradiation (Fig. 8b) after physical crosslinking was introduced. The agglomerated structures partially broke down after heat was applied (Fig. 8c). The full system snapshots are shown in Figure S5 in supporting information. Figure 8d shows the radial distribution function (RDF) of anthracene molecules under different simulating conditions. Highest agglomeration (blue curve) of anthracene was achieved after UV irradiation, which showed structures beyond third nearest neighbors, while the after-heating system (red line) showed weaker agglomeration with only 1st and 2nd nearest neighbors. The RDF in both the cases exhibited layered structures (equally spaced peaks). However, the layering is prominent in the case for the UV-irradiated system represented by larger sharp-peaks. Figure 8e compares the calculated RDF of anthracene molecules and flexible chains of the elastomers under different simulating conditions. In contrast to the well-defined structures (blue line) observed for the anthracene molecules after UV irradiation, the flexible chains exhibited an amorphous, liquid-like structure (green line), as indicated by only one RDF peak at 0.97 σ, corresponding to the monomer bead diameter, and no structure at longer length-scale. In addition, unlike the heat-induced scission of anthracene dimers (blue and red lines), no substantial structure change was observed for the flexible chains before and after heating (green and magenta lines), indicating its amorphous state.

**Figure 9:**
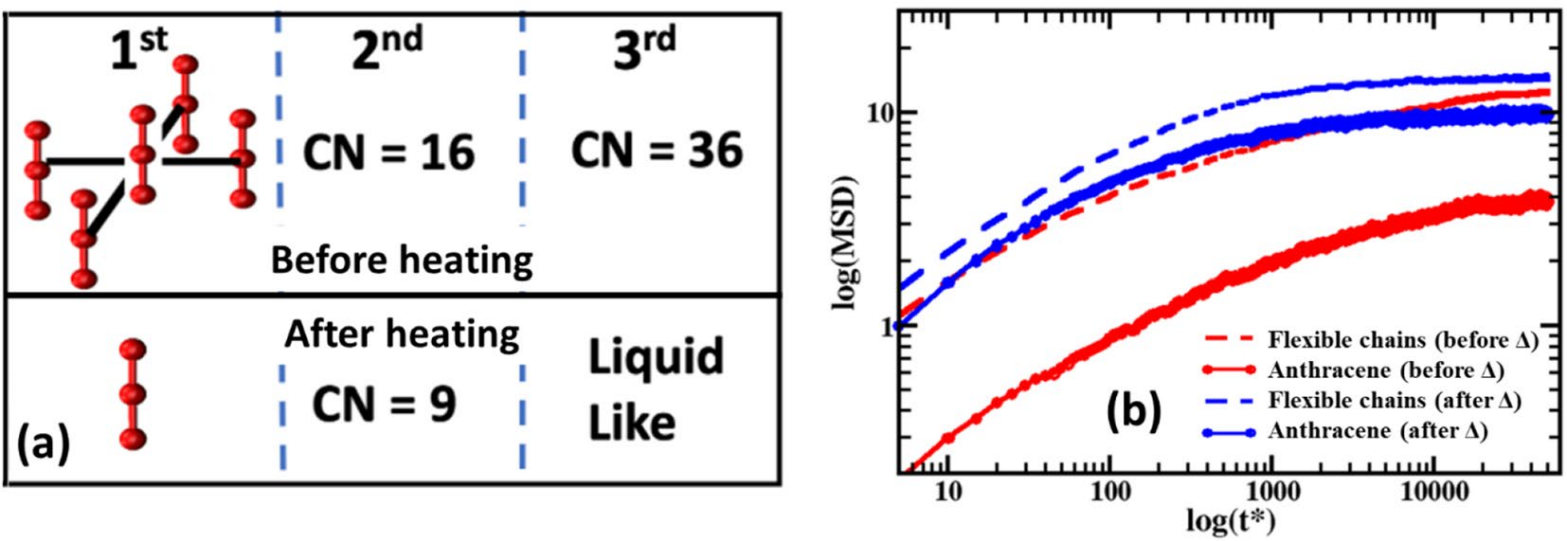
Structure and dynamics of anthracene molecules and flexible chains of the elastomers under different simulating conditions. (**a**) A pictorial representation of structure of anthracene molecules before and after heating based on peak position and coordination number obtained from RDF. (**b**) Mean-square-displacement of anthracene molecules and flexible chains before and after heating.

The heat-induced structure change of anthracene molecules was further investigated by comparing the coordination numbers (CN), as shown in Fig. 9a. Before heating, the anthracene molecules exhibited CN = 5.4, 16.2, and 36.2, corresponding to the three prominent peaks at r* = 1.15 σ, 1.95 σ, and 2.85 σ, respectively. A 1-D pictorial representation of CN is also shown in Fig. 9a. In the first coordination shell, five anthracene molecules are arranged in a 2-D simple cubic structure as shown in the top left panel. The middle bead of the central anthracene is coordinated with 6 other beads (CN = 6) with 2 from the central anthracene molecule and 4 from the neighboring anthracene molecules, all packed within 1.15 σ distance. The second coordination number (CN = 16) indicated a layered structure of this 2-D simple cubic. The third coordination number (CN = 36) indicated a well-populated third coordination shell. Therefore, we conclude that before heating (after UV treatment) the anthracene molecules formed tightly bonded layered agglomerated structures due to the strong interaction and crosslinking between the rigid molecules. In the experiment, it was observed that the chemically dimerized anthracene molecules resulted into agglomerated structures. The simulation, while using physical crosslinking, showed similar tightly bonded structures. From the above discussion we can infer that the size of these agglomerated structures is approximately 2.85 σ with 6 + 16 + 36 ~ 58 beads inside. Hence, there are 58/3 ~ 19 anthracene molecules in each cluster shown in Fig. 8b and the total estimated number of agglomerated clusters is 6000/19 ~ 300. The reversibility of these agglomerated structures can be observed after heating at 1.5 times of the initial temperature as shown in the RDF (Fig. 8d) and the bottom panel of Fig. 9a derived from CN. After heating, the CN of first and second coordination shells reduced to 2 and 9, corresponding to RDF peaks at 1.15 σ and 1.95 σ, respectively. The first coordination number CN = 2 indicated that after heating the agglomerated clusters of anthracene were broken up, resulting in individually scattered anthracene molecules in the system. However, the second coordination number CN = 9 indicated that some of the anthracene molecules were still not detached from the agglomerated structure.

The calculated mean-square-displacement (MSD) of anthracene molecules and flexible chains of the elastomers before and after heating is shown in Fig. 9b. It must be emphasized here that none of the MSD reached diffusive regime in this 5 million timesteps, which was due to the fact that our simulating system was a high-density melt (ρ* = 0.8) and hence overall motion was slowed down within the simulation timescale, representing an overall glassy system. However, this did not affect our investigation of the structures. As shown in Fig. 9b, before heating, anthracene molecules showed a lower MSD compared to that of flexible chains. The agglomerated state of the anthracene molecules restricted their motion, leading to a lower MSD. When heat was applied, a significant increase of MSD was observed for the anthracene molecules. As the anthracene dimers dissociated due to the applied heat, individual anthracene molecules became more mobile, resulting in an increased MSD. The dissociation of the anthracene dimers also led to a small increase of MSD of the flexible chains. However, the variation was not comparable to that of the anthracene molecules.

## Conclusions

We designed and synthesized anthracene-functionalized epoxy elastomers exhibiting combined light- and heat-induced shape memory behavior. Anthracene molecules were incorporated into the epoxy elastomer as pendent groups, allowing for a tunable network structure based on the reversible dimerization of the anthracene moiety. When exposed to 365 nm UV light, additional crosslinks were formed through cycloaddition of the anthracene molecules, leading to an increase of modulus and glass transition temperature, which was used as a mechanism for shape fixation. Exposure of the material to high temperatures resulted in the scission of the dimerized anthracene molecules, which reduced the modulus and glass transition temperature and allowed for shape recovery. Molecular dynamics simulation results indicated that dimerized anthracene molecules formed an aggregated structure with well-populated coordination shells. When exposed to heat, the agglomeration broken, and the system partially reversed to the initial state.

## Experimental

### Materials

Epichlorohydrin was obtained from Acros. BDE, 9-Anthracenemethanol, SA, and 1,5,7-triazabicyclo[4.4.0]dec-5-ene (TBD) were purchased from Sigma-Aldrich. Potassium hydroxide, tetrahydrofuran (THF), and ethanol were supplied by Fisher Scientific. All chemicals were used as received without further purification.

### Synthesis of AN epoxy monomer

9-Anthracenemethanol (5.0 g, 24 mmol), epichlorohydrin (11.1 g, 120 mmol), potassium hydroxide (2.69 g, 48 mmol), and THF (30 mL) were added in a 100 mL two-necked flask. The mixture was purged with nitrogen and refluxed for 24 h. After cooling to room temperature, the inorganic residue was filtered off and washed with THF. The filtrate was rotary evaporated under reduced pressure. The crude product was precipitated in deionized water and then purified by recrystallization in ethanol to afford the final product as a yellow solid (5.18 g, yield 83%). ^1^H NMR (400 MHz, CDCl_3_, δ): 8.48 (s, 1H), 8.40 (d, 2H), 8.03 (d, 2H), 7.56–7.48 (m, 4H), 5.63–5.54 (m, 2H), 3.95–3.91 (m, 1H), 3.64–3.60 (m, 1H), 3.23–3.22 (m, 1H), 2.81–2.78 (m, 1H), 2.65–2.63 (m, 1H). ^13^C NMR (400 MHz, CDCl_3_, δ): 131.40, 131.01, 129.00, 128.52, 128.29, 126.28, 124.95, 124.22, 70.67, 65.36, 51.12, 44.25. The synthesis route and NMR spectra of the AN epoxy monomer are shown in Figures S1–S3 in supporting information (SI).

### Preparation of epoxy elastomers

Epoxy monomers (AN and BDE) and curing agent (SA) with desired molar ratios were placed in a glass vial and were heated in an oil bath until fully melted. Then, TBD at an amount of 5 mol% of carboxylic acid groups was added as a ring-opening catalyst, followed by vigorous stirring for 1 min. Epoxy elastomer films were prepared using the parallel plate fixture of a strain-controlled rheometer (ARES G2, TA instruments). The plates were preheated to 170 °C before the mixture was loaded. The thickness (0.15 mm) of the epoxy films was controlled by adjusting the gap between the parallel plates. The samples were cured at 170 °C for 2 h using the rheometer. This method allowed for the preparation of films with uniform thickness and smooth surfaces and minimized uncertainties of DMA experiment. Epoxy elastomers with three different AN concentration (0, 20, and 40 mol%) were prepared and were referred to as EE-AN-0, EE-AN-20, and EE-AN-40, respectively.

### Preparation of thin epoxy films for UV–Vis measurement

The mixture of AN, BDE, SA, and TBD was drop-cast onto a quartz plate, then another quartz plate was placed onto it. To minimize the effect of oxygen in the UV–Vis experiment, the sample was cured between the two quartz plates. The mixture was heated to 170 °C and cured for 2 h. Unlike the bulk elastomers prepared using high concentration of AN, the sample used for the UV–Vis experiment contained 5 mol% of AN molecule and was labeled as EE-AN-5. The lower concentration of AN allowed for a better penetration of the incident light for UV–Vis measurements. The UV light exposure and UV–Vis measurements were performed while the sample was sandwiched between the quartz plates, during which the oxygen exposure was limited.

### Characterization

The chemical structure of the synthesized AN epoxy monomer was characterized using a Varian 400 MHz nuclear magnetic resonance (NMR) spectrometer at room temperature with deuterated chloroform (CDCl_3_) as the solvent and tetramethylsilane as the reference.

The reversible photo-crosslinking reaction of the epoxy elastomers with pendant anthracene groups was investigated using a UV–Vis spectrometer (Lambda 25, PerkinElmer). A thin layer of material with a thickness about 15 μm was prepared by drop-casting uncured mixture between two quartz plates (1″ × 1″ × 1/16″) followed by a thermal curing at 170 °C for 2 h. The epoxy film was irradiated by a 365 nm UV light with an intensity of 0.95 mW/cm^2^ for different time durations (0, 60, 180, 600, and 1200 s) using a handheld UV lamp (UVGL-55, Analytik Jena) and UV–Vis spectra were collected to study dimerization of the pendant anthracene group upon UV irradiation. Then, the material was irradiated by a 254 nm UV light with an intensity of 1.29 mW/cm^2^ for different time durations (0, 60, 180, and 600 s) using the same UV lamp and UV–Vis spectra were collected to investigate the scission of the dimerized anthracene groups.

The thermal properties of the epoxy elastomers were studied using a differential scanning calorimeter (Discovery DSC, TA Instruments) with a heat-cool-heat cycle at a ramp rate of 10 °C/min under nitrogen atmosphere. Glass transition temperature (T_g_) was determined from the midpoint of the step change in the second heating scan. The thermal stability of the materials was measured using a thermogravimetric analyzer (Discovery TGA, TA Instruments) under a nitrogen atmosphere with a heating rate of 10 °C/min. The dynamic mechanical properties of the as-synthesized UV-irradiated epoxy elastomers were investigated using the strain-controlled ARES G2 rheometer in dynamic mechanical analysis (DMA) mode with a film tension geometry. The dimension of the epoxy films was 25 mm × 3 mm × 0.15 mm. The films were tested from − 50 to 100 °C at a heating rate of 2 °C/min, a strain of 0.1%, and an oscillation frequency of 1 Hz. Crosslinking density (*μ*) of the elastomers was evaluated according to the equation *E*′ = 3*μRT*, where *E*’ is the rubbery modulus (storage modulus at T_g_ + 60 °C), *R* is the ideal gas constant (8.314 J/mol K), and *T* is the absolute temperature (K).

### Molecular dynamics simulations

The coarse-grained molecular dynamics (MD) simulations of the anthracene-based epoxy elastomers were performed using LAMMPS molecular dynamics package^35^. The model was generated based on a segment of the elastomeric network containing flexible chains, rigid pendant anthracene molecules, and physical crosslinking points (Figure S4a in SI). The CG polymer chain is a representation of the final polymer shown in the bottom panel of Fig. 2. Each repeating unit of the flexible chains was represented by a single bead of finite diameter, σ. All the beads, except those representing the anthracene groups, were bonded together by finite extensible non-linear elastic (FENE) spring following Kremer-Grest bead-spring model^36^. Anthracene molecules were represented by a rigid rod of three beads connected by harmonic bonds at 180º angle (Figure S4b in SI), typically done in simulation to represent stiff polymers. The flexible chains interacted via Lennard–Jones (LJ) potential. The intra-chain interactions were repulsive LJ except the crosslinking points, while the rigid anthracene groups were attractive to each other. The beads representing crosslink points were located at chain ends and a pendant end (Figure S4b in SI). Crosslinking of the modeled segments was performed using LAMMPS bond create technique that generates physical bonds of harmonic types between two monomers, which allowed for the formation of an infinite network structure for full system simulation^37^. The chain length or degree of polymerization before crosslinking was 44. We must emphasize here that ‘chemical reactions’ and hence chemical crosslinking in CG MD simulations is not possible. The only way to obtain crosslinking in CG MD is to create bonds between two or more monomer beads and physically bind them together with the same bond as the bond-forming polymer. This creates a large crosslinked network structure to of the crosslinked system without chemical reactions. The advantages of this technique are twofold, (1) it explains the fundamental physics of self-assembly and monomer dynamics qualitatively, and (2) it is computationally effective, especially for larger macromolecules such as the current system.

The initial system was constructed at a very high melt density (normalized number density, ρ* = 0.8 σ^−3^) by randomly filling the modeled network in a box of 69.0 × 69.0 × 69.0 σ^3^ (Figures S4c and S4d in SI). The system was equilibrated for 5 million steps at T* = 1.0, where T* = *k*_B_*T*/*ε* is the reduced temperature, *ε* is the LJ interaction strength, and *k*_B_ is the Boltzmann constant. During equilibration physical crosslinking by creating bonds between adjacent bond-forming monomers were turned on. We observed 98% crosslinking within first 1 million time-steps and hence this technique is highly efficient. The timestep for integration was, dt* = 0.01, where dt* is the LJ reduced time unit defined by $dt^{*} = dt \sqrt {\varepsilon /m\sigma^{2} }$, where *ε* and σ are the LJ interaction strength and bead diameter, respectively. Then, the interaction strength, e, between anthracene molecules was systematically increased to simulate the UV-triggered [4 + 4] cycloaddition of anthracene groups observed experimentally. The LJ ε parameter represents the strength between two monomers that does not represent chemical reactions but represent a mean-field number associated with all types of physical interactions between two monomers. The system was run for 5 million timesteps under this condition and structural and dynamic properties were calculated from an extra run of 5 million timesteps. Then, the system was heated to T* = 2.0 to entropically disordering the anthracene adducts to simulate the reverse cycloaddition process. However, after 1 million steps of simulation, it was found that T* = 2.0 resulted in an unstable simulation. Therefore, T* was reduced to 1.5, a temperature high enough to result in destabilization of the anthracene adduct. The system was run for 4 million more timesteps at T* = 1.5. The thermodynamic and dynamical properties were then obtained from an extra run of 5 million timesteps.

## Supplementary information

Supplementary Figures.

Supplementary Video S1.
