# Supplementary material for: Combined light- and heat-induced shape memory behavior of anthracene-based epoxy elastomers
Source: Sci Rep. 2020 Nov 19;10:20214. doi: 10.1038/s41598-020-77246-0 (PMC7677552; doi:10.1038/s41598-020-77246-0)
Supplement: Supplementary file 1 — Supplementary Figures. [file 41598_2020_77246_MOESM1_ESM.docx]

Supplementary Information

**Combined Light- and Heat-induced Shape Memory Behavior of Anthracene-based Epoxy Elastomers**

Yuzhan Li^1^, Monojoy Goswami^2^, Yuehong Zhang^3^, Tuan Liu^4^, Jinwen Zhang^4^, Michael R. Kessler^5^, Liwei Wang^4,*^ and Orlando Rios^6,*^

^1^ Energy and Transportation Science Division, Oak Ridge National Laboratory, Oak Ridge, TN 37831, United States

^2^ Chemical Sciences Division, Oak Ridge National Laboratory, Oak Ridge, TN 37831, United States

^3^ College of Bioresources Chemical and Materials Engineering, Shaanxi University of Science and Technology, Xi’an 710021, China

^4^ School of Mechanical and Materials Engineering, Washington State University, Pullman, WA 99164, United States

^5^ Department of Mechanical Engineering, North Dakota State University, Fargo, ND 58108, United States

^6^ Department of Materials Science and Engineering, The University of Tennessee, Knoxville, TN 37996, United States

* Corresponding authors:

Liwei Wang (Email: liwei.wang@wsu.edu) and Orlando Rios (Email: orios1@utk.edu).

**List of supplementary figures and videos**

Figure S1 Synthesis of 9-Anthracenemethoxyl glycidyl ether (AN)

Figure S2 ^1^H NMR spectrum of AN

Figure S3 ^13^C NMR spectrum of AN

Figure S4 Molecular dynamics simulation model description

Figure S5 Structure and dynamics of the elastomer under different simulating conditions

Video S1 Demonstration of light-induced shape fixation and heat-induced shape recovery

Figure S1. Synthesis route for AN epoxy monomer.


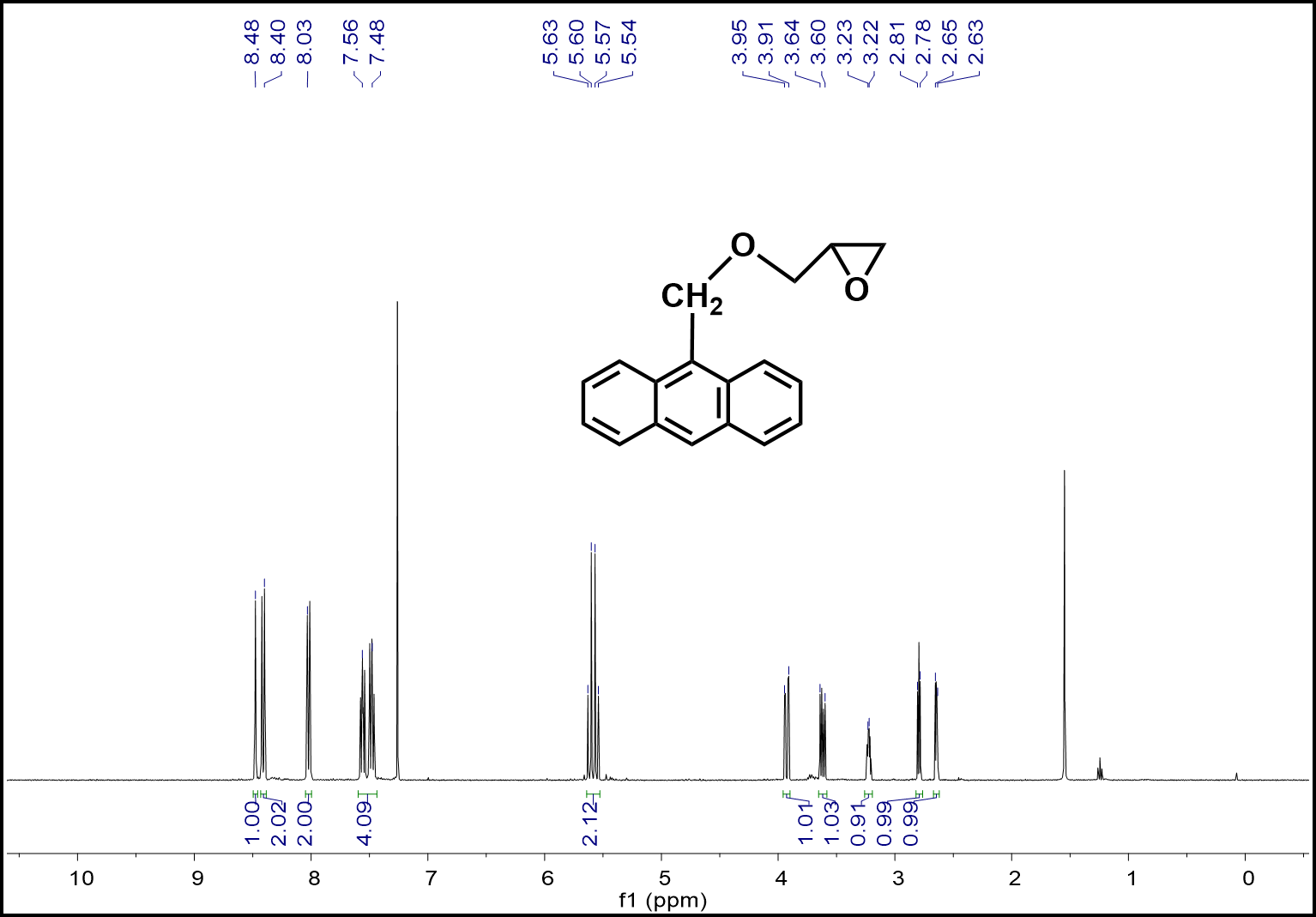


Figure S2. ^1^H NMR spectrum of the synthesized AN. ^1^H NMR (400 MHz, CDCl_3_, δ): 8.48 (s, 1H), 8.40 (d, 2H), 8.03 (d, 2H), 7.56−7.48 (m, 4H), 5.63−5.54 (m, 2H), 3.95−3.91 (m, 1H), 3.64−3.60 (m, 1H), 3.23−3.22 (m, 1H), 2.81−2.78 (m, 1H), 2.65−2.63 (m, 1H).


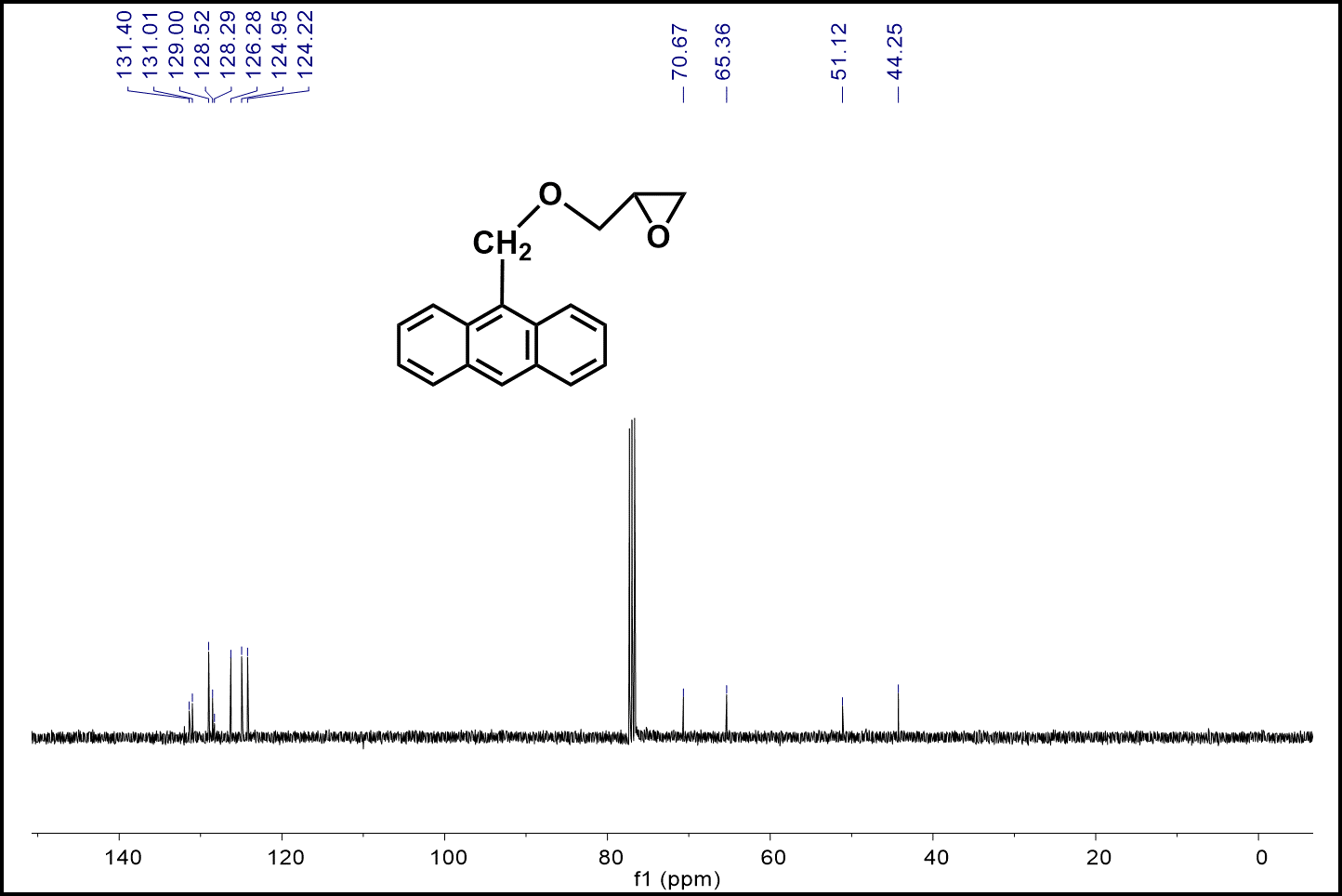


Figure S3. ^13^C NMR spectrum of the synthesized AN. ^13^C NMR (400 MHz, CDCl_3_, δ): 131.40, 131.01, 129.00, 128.52, 128.29, 126.28, 124.95, 124.22, 70.67, 65.36, 51.12, 44.25.


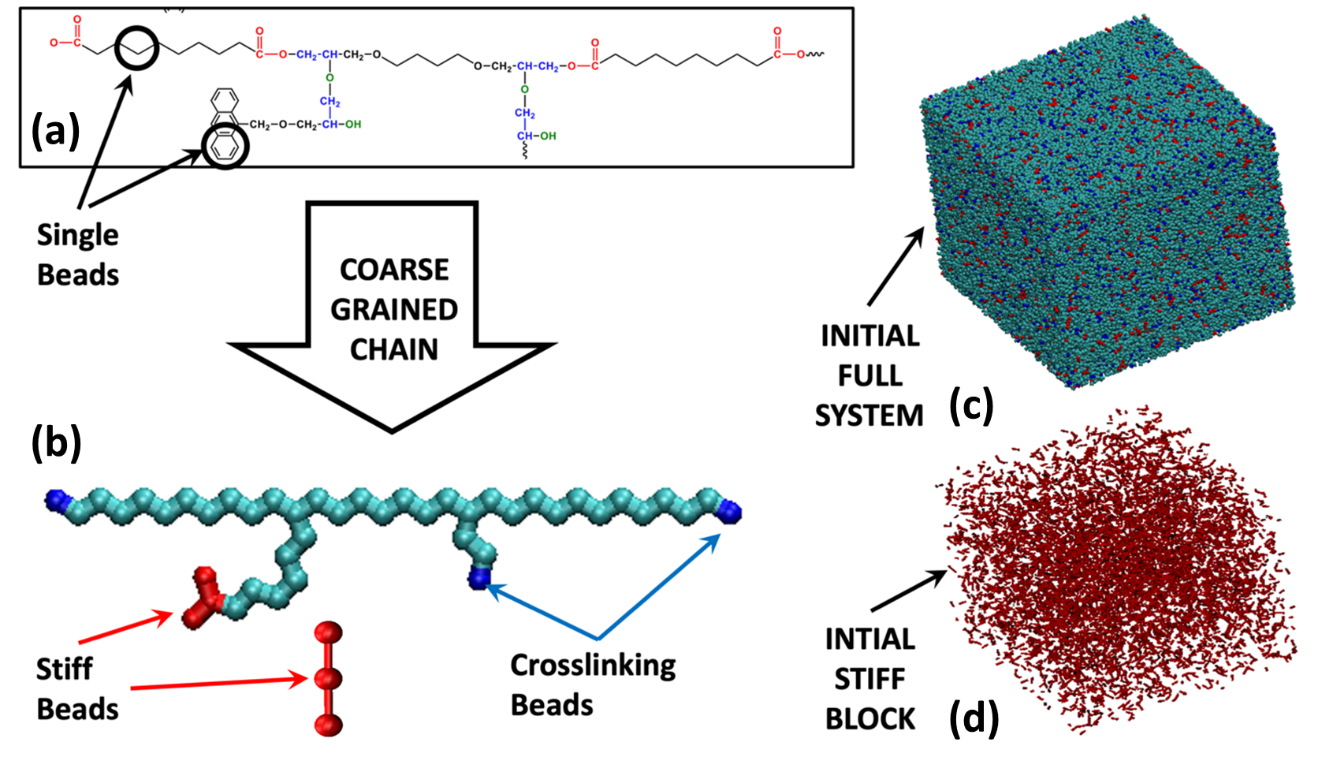


Figure S4. MD simulation model description. (a) Chemical structure of the epoxy elastomer segment containing rigid anthracene molecules and flexible chains. (b) Constructed coarse-grained elastomer segment. The model of the beads are shown in circles, the bead sizes are kept the same for computational simplicity. The crosslinking sites are shown in blue. The cyan color beads are flexible chains. The red beads are rigid anthracene pendant groups. (c) Snapshot of initial full system. (d) Snapshot of the anthracene molecules within the system.


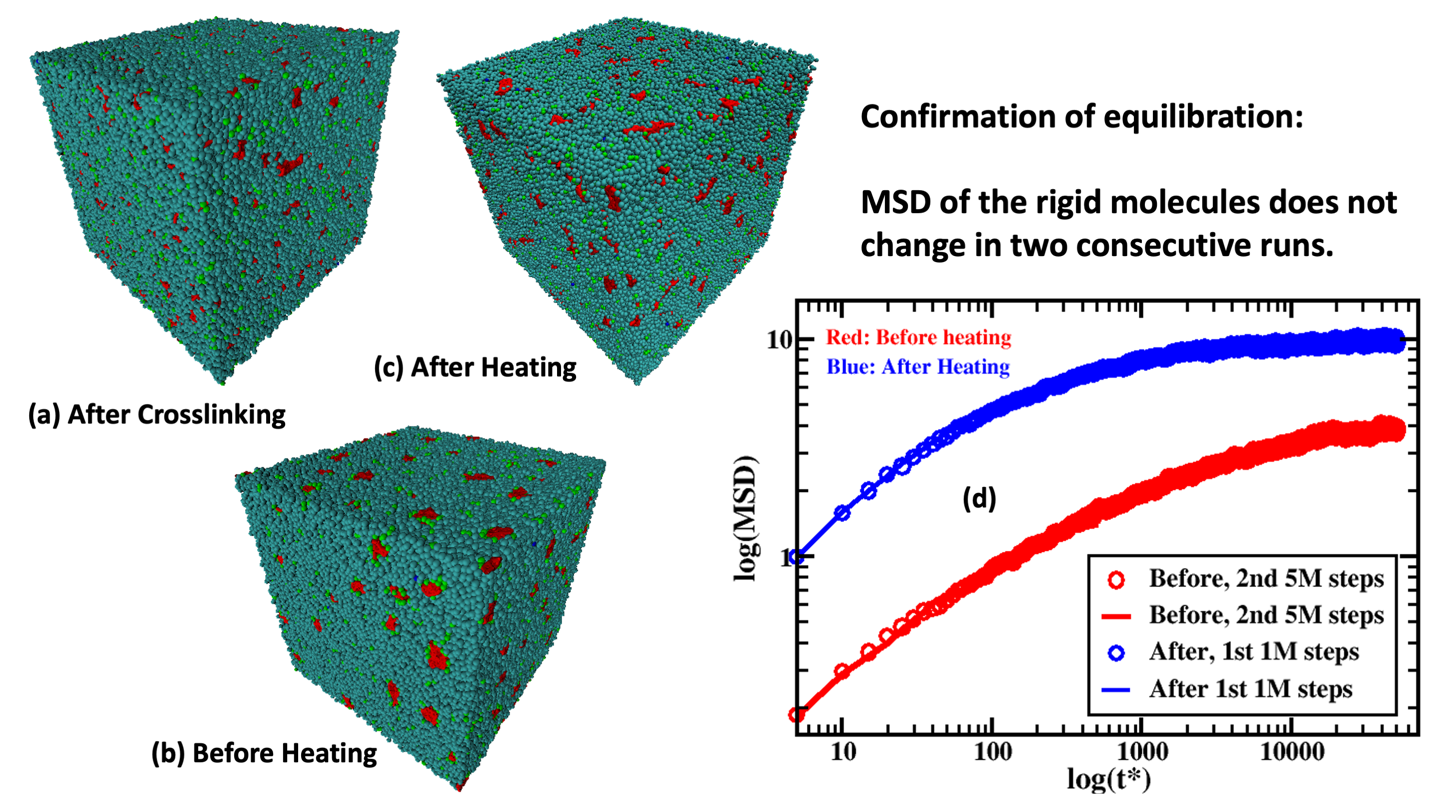


Figure S5. Structure and dynamics of the elastomer under different simulating conditions. (a) Snapshot of full system after crosslinking. (b) Snapshot of full system after UV irradiation but before heating. (c) snapshot of full system after heating. (d) Mean-square-displacement (MSD) for two consecutive runs one for 1M timesteps, and the second one for 5M timesteps. Both the MSD fell on each other indicating that the system was properly equilibrated.
